# Comparison of early and delayed removal of dressing following primary closure of clean and contaminated surgical wounds: A systematic review and meta-analysis of randomized controlled trials

**DOI:** 10.3892/etm.2020.8591

**Published:** 2020-03-11

**Authors:** Taijuan Zhang, Fujie Zhang, Zongnan Chen, Xiuling Cheng

**Affiliations:** 1 Wound Treatment Center, Tianjin 300450, P.R. China; 2 Department of General Surgery, Tianjin Fifth Central Hospital, Tianjin 300450, P.R. China; 3 Department of Nursing, Tianjin Fifth Central Hospital, Tianjin 300450, P.R. China

**Keywords:** occlusive dressings, surgical wound, wounds and injuries, wound closure techniques

## Abstract

The usefulness of dressing a surgical wound beyond the first 24-48 h of surgery is currently a controversial issue. The aim of this meta-analysis was to compare the early and delayed removal of dressing following primary closure in the management of clean and contaminated surgical wounds. Systematic searches were conducted in various databases including Medline, Cochrane Controlled Register of Trials (CENTRAL), Scopus, and Embase from January, 1964 until October, 2019. We used the Cochrane risk of bias tool to assess the quality of published trials. We carried out a meta-analysis with random-effects model and reported pooled risk ratios (RR) with 95% confidence intervals (CIs). In total, we analysed 10 studies with 1,708 participants. All the studies were randomized controlled trials, while the majority of studies had unclear or high bias risks. Early dressing removal was favoured with respect to surgical site infection (pooled RR=0.89; 95% CI: 0.61 to 1.29), patient's perception on safety (pooled RR=0.60; 95% CI: 0.48 to 0.76) and comfort (pooled RR=0.95; 95% CI: 0.74 to 1.22), while the remaining outcomes favoured delayed dressing removal. However, none of the factors had statistically significant difference between two interventions except the patient's perception on safety. To summarize, delayed removal of dressing is not superior to early removal following primary closure of clean or clean-contaminated surgical wounds.

## Introduction

Most of the surgical procedures involve an incision in the skin to allow the surgeon to gain access to the deeper organs or tissues. Most of these wounds are fully closed (primary closure) at the end of the surgery (1). These wounds are covered by the surgeon using either adhesive tape or dressing (2-4). Use of dressing can act as a barrier from infection and protect the wound until the restoration of skin continuity (5). It can also absorb the exudate from the wound, keeps it dry, clean, and prevents bacterial contamination from the external environment (6-8). Ideally, surgeons should select appropriate dressings in order to ensure the wound remains free of excessive slough, toxic chemicals, and infection and remain at optimal temperature and pH for healing.

Post-surgical wound dressings are usually left at the site for at least 48 h or until suture removal (delayed removal of dressing), irrespective of contamination level of the wounds, or other factors such as antibiotic administration. Previous findings have shown that the moist environment formed by the dressings accelerates the wound healing process (9). However, other findings suggest that this can be a disadvantage, as excess exudate causes softening and deterioration of wound and surrounding healthy tissues (10). Delayed removal can cause inconvenience and dissatisfaction among the patients and increase length of nursing time, which ultimately lead to an increase in economic burden (11-13). In addition, dressing increases the chance of local sweating and reduces the moisture evaporation. This results in higher dampness which potentially acts as a point of entry for microorganisms for infection (14).

The usefulness of dressing a surgical wound beyond the first 24-48 h of surgery is therefore controversial. Thus, the aim of this meta-analysis was to assess the comparative efficacy, in terms of surgical site infection, wound dehiscence and patient perception, as well as satisfaction between early removal and delayed removal of dressing following primary closure in the management of clean and contaminated surgical wounds.

## Materials and methods

### 

#### Inclusion criteria for the review

#### Type of studies to be included

We included parallel arm individual randomized, quasi-randomized or cluster-randomized controlled trials for the present review. Studies reported as full text were included while studies published with only the abstract or unpublished data were excluded.

#### Type of participants

We included studies conducted among patients with clean and contaminated surgical wound, irrespective of the type of surgery.

#### Type of intervention

We included studies that directly compared the early and delayed removal of dressing following primary closure irrespective of the duration of early or delay in removal.

#### Type of outcome measure

Outcome measures were then assessed: Surgical site infection, wound dehiscence, patient satisfaction and patient's perception on safety, comfort, dehiscence. We included the studies reporting any of the outcomes mentioned above in both the arms.

#### Search strategy

We conducted an extensive electronic search in the following databases: Medline, Scopus, Embase and Cochrane Central Register of Controlled Trials (CENTRAL), clinical trial registries such as the ClinicalTrials.gov and WHO International Clinical Trials Registry Platform (ICTRP). Combination of medical subject heading (MeSH) and free text terms were used for carrying out a literature search. The MeSH terms ‘dressing wounds’, ‘clean and contaminated wound’, ‘dressing removal’, ‘early dressing removal’, ‘delayed dressing removal’, ‘surgical wounds’ and ‘randomized controlled trial’ along with free text terms were used in all search engines for the above-mentioned databases in various combinations. The search was conducted in all the databases from January, 1964 to October, 2019 with publication language restricted to English.

#### Searching other resources

We checked the reference list of primary trials obtained through electronic search, and relevant articles were included in the review and analysis. We contacted the authors of the published trials in case clarification or additional information was required for the methodological assessment of the studies included.

#### Data collection and analysis

#### Selection of studies

Two independent investigators independently performed a literature search and screened the title, abstract and keywords of all the studies identified for possible inclusion in the review. Full-text articles were obtained for studies considered to be relevant. Further screening of the abstract and full text of the retrieved articles was performed independently by primary and secondary investigators to select the studies that satisfied the eligibility criteria of the present review. Any disagreements during the entire selection process between two investigators were resolved either through consensus or consultation with a third investigator. Quality of the overall review process was monitored by the third investigator. The preferred Reporting Items for Systematic Review and Meta-Analysis (PRISMA) check list were used for reporting of the present review (http://prisma-statement.org/prismastatement/Checklist.aspx).

#### Data extraction and management

The primary investigator extracted the relevant study characteristics for the review from the included studies. The data extracted were: General information such as date of extraction; study title and authors; details under methods section such as study design, participants and study setting; details under participants' section such as total number of participants in each arm, baseline and endline outcome measures, inclusion and exclusion criteria; details under interventions section such as details of intervention group, details of comparison group and follow up duration; details under outcome section such as primary and secondary outcomes captured in the study and time of outcome assessment and other details necessary for assessing the quality of studies.

Primary and secondary investigators independently extracted data related to outcome measure from the included studies. When studies report multiple arms in a single trial, only the relevant arms were included for the analysis. The primary investigator transferred the obtained data into the statistical software RevMan (ver 5.3). Data entry was double checked for correct entry by the third investigator through comparison of data presented in review and included study reports.

#### Risk of bias assessment in included studies

Two independent investigators assessed the risk of bias for included studies using Cochrane risk of bias tool for RCTs (https://handbook-51.cochrane.org/chapter_8/8_assessing_risk_of_bias_in_included_studies.htm). The domains used for assessing the risk of bias were: Random sequence generation, allocation concealment, blinding of the participants, incomplete outcome data, blinding of outcome assessment and selective reporting of outcome. For each of the abovementioned domains, risk of bias was graded as low (if adequate information was provided), high (if the information was inadequate or not performed) and unclear (if the information was missing).

#### Statistical analysis

Meta-analysis was performed with the selected studies using RevMan 5·3 (Copenhagen: The Nordic Cochrane Centre, The Cochrane Collaboration, 2014). Since all the outcomes were dichotomous, the number of events and participants in each group were obtained and entered into the Revman software to estimate the pooled effect size in terms of relative risk. We used the random effects model with inverse variance. In case of missing data, the author of the included trial was contacted and the necessary data could still not be retrieved, the imputation method was followed.

#### Assessment of heterogeneity

Evidence of between-study variance due to heterogeneity was assessed through the Chi-square test of heterogeneity and I^2^ statistics to quantify the inconsistency. I^2^<25% was mild, 25-75% was moderate and >75% was considered as substantial heterogeneity. Study specific and pooled estimates were graphically represented through forest plot.

#### Assessment of reporting biases

Reporting bias was assessed by checking whether the included trial is registered in a trial registry or full protocol is available. If available, list of outcomes in the protocol were compared with the list of outcomes mentioned in the full published trial. Publication bias was assessed using Egger's test and graphically represented by the funnel plot.

#### Subgroup analysis and investigation of heterogeneity

There was no significant heterogeneity for primary outcome on surgical site infection, while rest of the outcomes did not have sufficient number of studies to perform subgroup analysis or meta regression.

## Results

### 

#### Selection of studies

**Figure 1:**
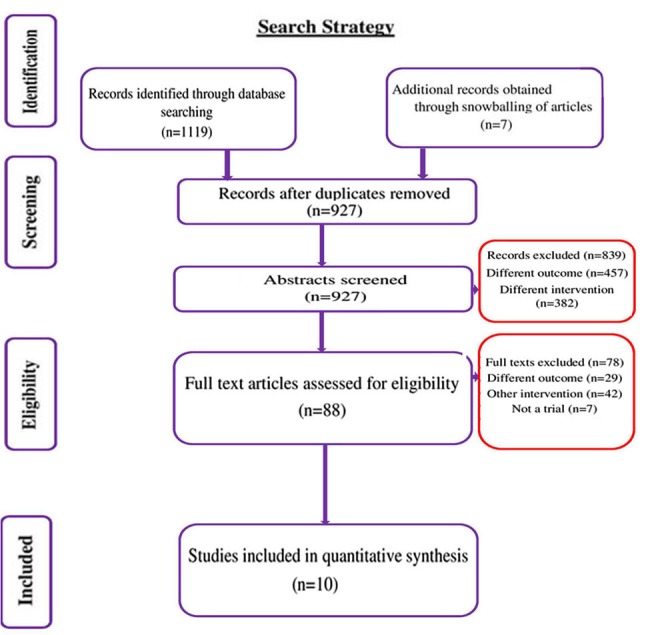
PRISMA flow chart showing the selection of studies for the present review (n=10).

We conducted a systematic search to identify studies that directly compared the early vs. delayed removal of dressing following primary closure of surgical wound from January, 1964 until October, 2019. In total, 1,119 citations were identified, of which 522 trials were retrieved from Medline, 242 from Scopus, 232 from Embase, 111 from CENTRAL, 7 from ClinicalTrials.gov and 5 from WHO ICTRP. After the first stage of screening (title, abstract and keywords), 88 relevant studies were retrieved. Full text of these studies was reviewed for eligibility criteria. Bibliographies of the retrieved articles were reviewed and 7 relevant studies were retrieved. Finally, 10 studies with 1,708 participants satisfying the inclusion criteria were included (Fig. 1) (13,15-23).

#### Characteristics of studies included

Characteristics of the studies are described in Table I. All the included studies were RCTs. Most the studies (7 out of 10) were conducted in countries belonging to American regions including the USA and Brazil. In total, 1,708 participants were found in the included studies with 853 participants in the early dressing removal arm and 855 participants in the delayed removal arm. Sample size (both arms together) varied from 70 to 602 while sample size in intervention arm varied from 35 to 300 and in control arm varied from 35 to 302. Among the 10 studies included, all the studies reported on surgical site infection, 4 studies reported on wound dehiscence, 2 studies reported on patient satisfaction, patient's perception on safety, comfort and convenience.

#### Methodological quality of the included studies

Assessment of risk of bias is done for RCTs (Table II). Most of the studies had low risk of bias with respect to bias arising from randomization process (random sequence generation and allocation concealment). All included studies had either high or unclear risk of bias with respect to blinding of participants. Three of 10 included studies had low risk of bias with respect to blinding of outcome assessment. All studies had high or unclear risk of bias with respect to incomplete outcome data except Peleg *et al* (18). All studies had high or unclear risk of bias with respect to selective reporting of outcome except Ramkumar *et al* (19).

#### Surgical site infection (SSI)

**Figure 2:**
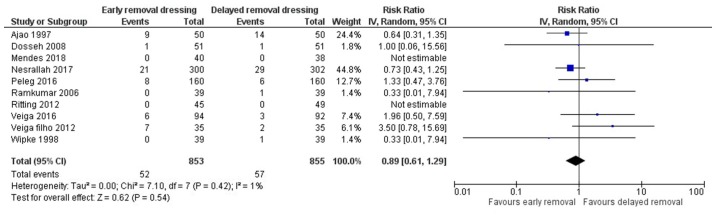
Forest plot showing the difference in surgical site infection between early and delayed dressing removal techniques (n=10).

**Figure 3:**
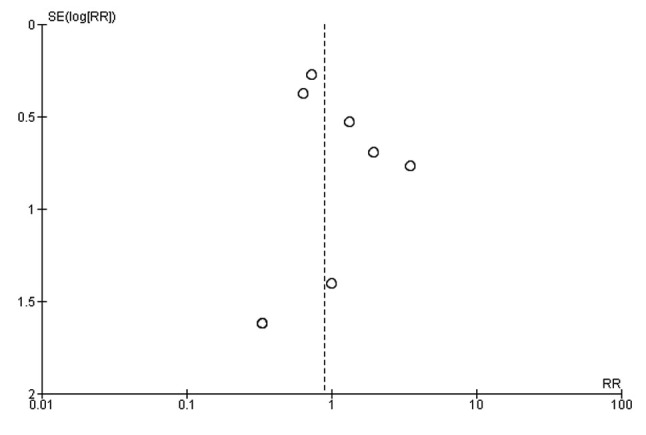
Funnel plot checking for publication bias (n=10).

All 10 included studies reported on the surgical site infection in both arms (13,15-23). The pooled RR was 0.89 (95% CI: 0.61 to 1.29) (Fig. 2). This indicates that the patients having early removal of dressing have 11% less risk of having SSI when compared to patients having delayed removal of dressing following surgery. However, this association was not statistically significant (P=0.54). There was no heterogeneity among the studies reporting surgical site infections (I^2^=1%, P=0.42). Funnel plot showed a symmetrical plot indicating absence of publication bias (Fig. 3). Egger's test also confirmed the finding with P-value of 0.30 indicating the absence of small study effects.

#### Wound dehiscence

**Figure 4:**
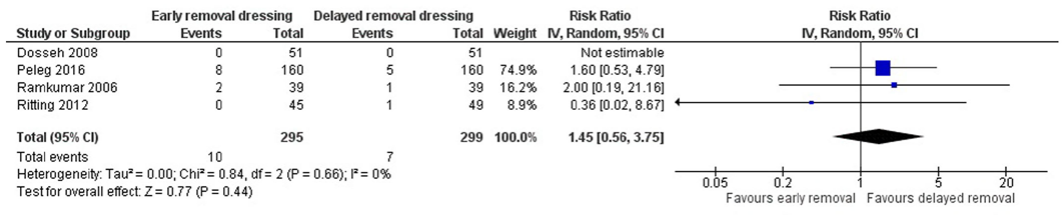
Forest plot showing the difference in surgical wound dehiscence between early and delayed dressing removal techniques (n=4).

Four studies have reported on the wound dehiscence in both the arms (13,18-20). The pooled RR was 1.45 (95% CI: 0.56 to 3.75) (Fig. 4). This indicates that the patients having early removal of dressing have 1.45-fold higher risk of having wound dehiscence when compared to patients having delayed removal of dressing following surgery. However, this association was not statistically significant (P=0.44). There was no heterogeneity among the studies reporting the wound dehiscence (I^2^=0%, P=0.66).

#### Patient satisfaction

**Figure 5:**
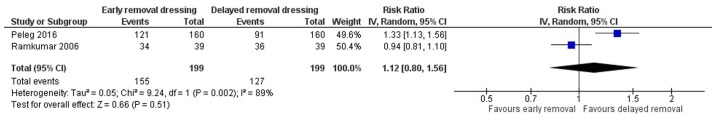
Forest plot showing the difference in patient satisfaction between early and delayed dressing removal techniques (n=2).

Two studies have reported on the patient satisfaction in both the arms (18,19). The pooled RR was 1.12 (95% CI: 0.80 to 1.56) (Fig. 5). This indicates that the patients having early removal of dressing have 1.12 times higher chance of being satisfied when compared to patients having delayed removal of dressing following surgery. However, this association was not statistically significant (P=0.51). There was a significant heterogeneity among the studies reporting patient satisfaction (I^2^=89%, P=0.002).

#### Patient's perception on safety

**Figure 6:**
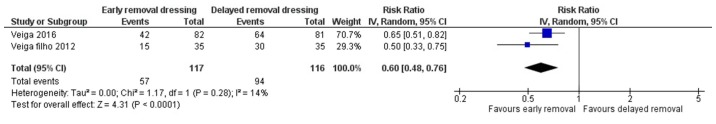
Forest plot showing the difference in patient perception on safety between early and delayed dressing removal techniques (n=2).

Two studies have reported on the patient's perception on safety following early or delayed removal of dressing for surgical wounds (21,22). The pooled RR was 0.60 (95% CI: 0.48 to 0.76) (Fig. 6). This indicates that the patients having early removal of dressing have 40% higher perception of being safe when compared to patients having delayed removal of dressing following surgery. This association was found to be statistically significant (P<0.001). There was no heterogeneity among the studies reporting patient's perception on safety (I^2^=14%, P=0.28).

#### Patient's perception on comfort

**Figure 7:**
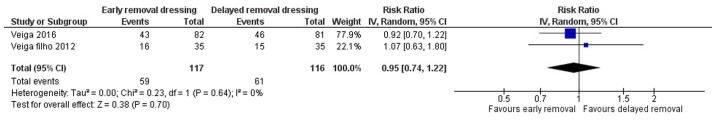
Forest plot showing the difference in patient perception on comfort between early and delayed dressing removal techniques (n=2).

Two studies have reported on the patient's perception on comfort following early or delayed removal of dressing for surgical wounds (21,22). The pooled RR was 0.95 (95% CI: 0.74 to 1.22) (Fig. 7). This shows that there is no significant difference in patient's perception on comfort following early or delayed removal of dressing (P=0.70). There was no heterogeneity among the studies reporting patient's perception on comfort (I^2^=0%, P=0.64).

#### Patient's perception on convenience

**Figure 8:**
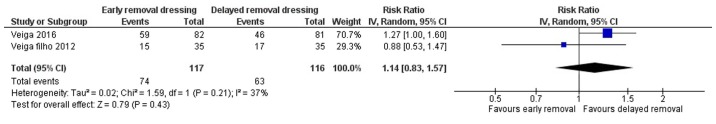
Forest plot showing the difference in patient perception on convenience between early and delayed dressing removal techniques (n=2).

Two studies have reported on the patient's perception on convenience following early or delayed removal of dressing for surgical wounds (21,22). The pooled RR was 1.14 (95% CI: 0.83 to 1.57) (Fig. 8). This shows that there is no significant difference in patient's perception on convenience following early or delayed removal of dressing (P=0.43). There was moderate heterogeneity among the studies reporting patient's perception on convenience (I^2^=37%, P=0.21).

## Discussion

Dressings put on surgical wounds following primary closure can be removed early or delayed (retained) until the suture is removed or strips. However, we could not find any review comprehensively assessing the effect of early or delayed removal of dressing following surgery in reducing surgical site infection, wound dehiscence or patient’s satisfaction or perception. Thus, this review was conducted with an objective of comparing the early with delayed removal of dressing following primary closure of surgical wound in terms of clinical and quality outcomes among patients undergoing surgery. We tried to compile the best possible evidence currently available.

In total, we identified 10 studies with 1,708 participants for our analysis. All the included studies were RCTs. The majority of the studies were conducted in countries of the American regions including the USA and Brazil. Most of the included studies had high or unclear risk of bias with respect to all the domains except randomization process domains. We did not find any substantial heterogeneity for most of the outcomes in the studies except studies reporting patient satisfaction. Nonetheless, we did not have an adequate number of studies to perform a subgroup analysis or meta-regression to explore the source of heterogeneity for the studies reporting patient satisfaction.

Clinical outcome such as SSI and patient perception on safety and comfort favoured the early dressing removal arm while outcomes such as wound dehiscence, patient satisfaction and patient perception on convenience favoured the delayed dressing removal arm. However, conclusive or significant evidence was found only for patient perception on safety which favoured early removal of dressing. For all other outcomes, we did not find conclusive or significant evidence for any of these outcomes as the confidence limit crossed the null value in all the outcomes assessed. This shows that timing of removal of dressing following surgical wound does not have significant impact on clinical outcomes or patient perception or satisfaction. One similar review was conducted before on this topic, a meta-analysis by Toon *et al* which compared early and delayed removal of dressing, reported almost similar findings to our review (24). However, the previous review included only 4 trials and outcomes related to patient satisfaction and patient perception were not assessed. The present review includes 10 studies and reported these additional outcomes. This should be useful in making better decisions and judgement in choosing the timing of removal of dressing for the patients following primary closure of surgical wounds.

The major strengths of our study include the comprehensive search of literature and the broad search strategy to gather all the required publications up-to-date. Our review adds to the limited evidence available on direct comparison of the early and delayed removal of dressing for the management of patients with postoperative surgical wounds. We only included RCTs in our review which enables us to infer causal associations between intervention and outcomes. We also included patient's perception and satisfaction as this may provide added advantage while taking informed decisions on timing of removal of dressing for surgical wounds. We also assessed publication bias for the main outcome on SSI and found almost symmetrical funnel plot. Nevertheless, there are limitations to our review. We could not assess the source of heterogeneity for outcomes that showed significant heterogeneity due to the limited number of studies. Finally, most of the studies included in our review were conducted in American regions, which may limit the generalizability of our findings to other geographical regions.

Our study has certain implications towards clinical practice. There is a sense of uncertainty and inconsistency revolving around the timing of removal of dressing following primary closure in surgery. Our study may be useful in overcoming this sense of inconsistency and across the findings and provided a reliable pooled estimate for the same. We found that delayed removal of dressing did not have significantly better clinical outcomes when compared to early removal of dressing in the management of surgical wounds. Early removal of dressing from clean/clean contaminated surgical wounds seems to have no negative effect on postoperative patients. Furthermore, patients seem to have the perception that early removal of dressing is safer when compared to delayed removal. However, these findings are based on studies with high or unclear risk of bias and can be applied to patients with surgical wounds closed by primary intention. In addition, the surgical wound healing process is related to the type of dressing applied (25). Evidences have shown that honey dressing has significantly faster wound healing compared to any other type of dressings (26,27). Thus, the nature and type of dressing should also be taken into account before deciding the timing of removal of dressings for any surgical wounds. Application of these findings to patients with accidental injuries or delayed primary closure is unclear. In addition, none of the studies have reported on health-related quality of life of the patients. Thus, further high-quality trials with a focus on quality of life component have to be conducted in the future.

In conclusion, delayed removal of dressing is not superior to early removal following primary closure of clean or clean-contaminated surgical wounds. However, more robust RCTs with large sample size are required to derive conclusive evidence towards health-related quality of life and applicability of these findings for delayed primary closure or accidental injuries.

## Figures and Tables

**Table I:** Characteristics of the included studies, n=10.

No	Author, year (Refs.)	Country	Study design	Type of Surgery	Sample size in early removal arm	Sample size in delayed removal arm	Intervention	Mean age
1	Ajao 1997(15)	Nigeria	Randomized controlled trial	Various surgical procedures (all operations on the trunk)	50	50	Group 1 (n=50): Early dressing removal, wound left open 24-36 h after suturing. Group 2 (n=50): Delayed dressing removal, dressing left for 7-10 days unless infection suspected, when the wound was inspected and dressing reapplied.	Not provided
2	Dosseh *et al*, 2008(13)	Togo	Randomized controlled trial	Abdominal surgery, neck surgery and thoracic surgery and having clean or clean-contaminated wounds	51	51	Group 1 (n=51): Early dressing removal,wound left open 48 h after surgery. Group 2 (n=51): Delayed dressing removal, dressing changed every 48 h until suture removal.	36 years Group A=26 years Group B=27.5 years
3	Mendes *et al*, 2018(16)	Brazil	Randomized controlled trial	Breast augmentation surgery	40	38	Group 1 (n=40): Early dressing removal, wound left open 24 h after surgery.	Group 2 (n=38): Delayed dressing removal, ressing left for 6 postoperative days.
4.	Nesrallah *et al*, 2017(17)	USA	Randomized controlled trial	Caesarean section	300	302	Group 1 (n=300): Early dressing removal, wound left open 12-30 h after surgery. Group 2 (n=302): Delayed dressing removal, dressing left for 30-48 h after surgery	Not provided
5	Peleg *et al*, 2016(18)	USA	Randomized controlled trial	Caesarean section	160	160	Group 1 (n=160): Early dressing removal, wound left open 6 h after surgeryGroup 2 (n=160): Delayed dressing removal, dressing left for 24 h after surgery	Group A=32.9 years Group B=31.6 years
6	Ramkumar *et al*, 2006(19)	UK	Randomized controlled trial	Unilateral or bilateral primary correction of prominent ears	39	39	Group 1 (n=39): Early dressing removal, head bandage was removed the next day. Group 2 (n=39): Delayed dressing removal, head bandage was removed after 10 days. Both groups received Tubigrip bandage at night-time for 4-6 weeks	10 years
7	Ritting *et al*, 2012(20)	USA	Randomized controlled trial	Mini-open carpal tunnel release	45	49	Group 1 (n=45): Early dressing removal, within 48-72 h. Group 2 (n=49): Delayed dressing removal, after 2 weeks.	Group A = 46.3 years Group B = 44.8 years
8	Veiga *et al*, 2016(21)	Brazil	Randomized controlled trial	Breast reconstruction surgery	94	92	Group 1 (n=94): Early dressing removal, within 24 h. Group 2 (n=92): Delayed dressing removal, after 6 days.	Group A = 47.8 years Group B = 49.3 years
9	Veiga-Filho *et al*, 2012(22)	Brazil	Randomized controlled trial	Reduction mammoplasty	35	35	Group 1 (n=94): Early dressing removal, within 24 hours Group 2 (n=92): Delayed dressing removal, after 6 days.	34 years
10	Wipke-Tevis and Stotts, 1998(23)	USA	Randomized controlled trial	Coronary artery bypass graft surgery with saphenous vein grafts	39	39	Group 1 (n=39): Early dressing removal, wound left open 24 h after surgery Group 2 (n=39): Delayed dressing removal, dressing remained in place until removal of sutures.	62 years

**Table II:** Risk of bias assessment for the included studies, n=10.

No	Author year, (Ref)	Random sequence generation	Allocation concealment	Blinding of the participants	Blinding of outcome assessment	Incomplete outcome data	Selective reporting of outcome
1	Ajao 1997, (15)	Unclear risk	Unclear risk	High risk	Unclear risk	Unclear risk	High risk
2	Dosseh *et al*, 2008(13)	Unclear risk	Unclear risk	High risk	Unclear risk	High risk	High risk
3	Mendes *et al*, 2018(16)	Low risk	Low risk	Unclear risk	Unclear risk	High risk	Unclear risk
4	Nesrallah *et al*, 2017(17)	Unclear risk	Unclear risk	High risk	High risk	Unclear risk	Unclear risk
5	Peleg *et al*, 2016(18)	Low risk	Low risk	High risk	High risk	Low risk	Unclear risk
6	Ramkumar *et al*, 2006(19)	Unclear risk	Unclear risk	High risk	Unclear risk	High risk	Low risk
7	Ritting *et al*, 2012(20)	Low risk	Unclear risk	High risk	Low risk	High risk	Unclear risk
8	Veiga *et al*, 2016(21)	Low risk	Low risk	High risk	Low risk	High risk	Unclear risk
9	Veiga-Filho *et al*, 2012(22)	Low risk	Low risk	High risk	Low risk	High risk	Unclear risk
10	Wipke-Tevis and Stotts, 1998(23)	Low risk	Low risk	High risk	Unclear risk	High risk	High risk

## Data Availability

The datasets used and/or analyzed during the current study are available from the corresponding author on reasonable request.
